# Association of SARS-CoV-2 Nucleocapsid Protein Mutations with Patient Demographic and Clinical Characteristics during the Delta and Omicron Waves

**DOI:** 10.3390/microorganisms11051288

**Published:** 2023-05-15

**Authors:** Feda A. Alsuwairi, Asma N. Alsaleh, Madain S. Alsanea, Ahmed A. Al-Qahtani, Dalia Obeid, Reem S. Almaghrabi, Basma M. Alahideb, Maha A. AlAbdulkareem, Maysoon S. Mutabagani, Sahar I. Althawadi, Sara A. Altamimi, Abeer N. Alshukairi, Fatimah S. Alhamlan

**Affiliations:** 1 Department of Infection and Immunity, King Faisal Specialist Hospital and Research Center, Riyadh 11211, Saudi Arabia; 2 Botany and Microbiology Department, College of Science, King Saud University, Riyadh 11451, Saudi Arabia; 3 College of Medicine, Alfaisal University, Riyadh 11533, Saudi Arabia; 4 Public Health Laboratories, Public Health Authority, Riyadh 13351, Saudi Arabia; 5 Organ Transplant Center of Excellence, King Faisal Specialist Hospital and Research Center, Riyadh 11211, Saudi Arabia; 6 Department of Pathology and Laboratory Medicine, King Faisal Specialist Hospital and Research Center, Riyadh 11211, Saudi Arabia; 7 Department of Medicine, King Faisal Specialist Hospital and Research Center, Jeddah 23433, Saudi Arabia

**Keywords:** SARS-CoV-2, COVID-19, Delta, Omicron, nucleocapsid (N) protein, mutation

## Abstract

SARS-CoV-2 genomic mutations outside the spike protein that may increase transmissibility and disease severity have not been well characterized. This study identified mutations in the nucleocapsid protein and their possible association with patient characteristics. We analyzed 695 samples from patients with confirmed COVID-19 in Saudi Arabia between 1 April 2021, and 30 April 2022. Nucleocapsid protein mutations were identified through whole genome sequencing. 𝜒^2^ tests and *t* tests assessed associations between mutations and patient characteristics. Logistic regression estimated the risk of intensive care unit (ICU) admission or death. Of the 60 mutations identified, R203K was the most common, followed by G204R, P13L, E31del, R32del, and S33del. These mutations were associated with reduced risk of ICU admission. P13L, E31del, R32del, and S33del were also associated with reduced risk of death. By contrast, D63G, R203M, and D377Y were associated with increased risk of ICU admission. Most mutations were detected in the SR-rich region, which was associated with low risk of death. The C-tail and central linker regions were associated with increased risk of ICU admission, whereas the N-arm region was associated with reduced ICU admission risk. Consequently, mutations in the N protein must be observed, as they may exacerbate viral infection and disease severity. Additional research is needed to validate the mutations’ associations with clinical outcomes.

## 1. Introduction

Coronavirus disease 2019 (COVID-19) is a respiratory disease presenting a global health threat. The disease usually spreads through direct exposure to the infectious particles in respiratory droplets and bioaerosol particles. The COVID-19 pandemic was first reported in Wuhan, China, in December 2019. The pandemic rapidly moved to Iran, Thailand, Japan, South Korea, and Singapore. After the initial few months of 2020, the virus expanded to the United States, the United Arab Emirates, the United Kingdom, Italy, and Spain. Subsequently, the outbreak of COVID-19 was announced to be a pandemic by the World Health Organization (WHO) on 11 March 2020 [1]. Severe acute respiratory syndrome coronavirus 2 (SARS-CoV-2) is the etiological agent of COVID-19. It is an RNA virus, which tends to accumulate mutations in the genome. Various mutations promoted the development of new virus variants [2]. Thus, several global efforts have been devoted to tracking changes in the genome. The World Health Organization (https://www.who.int/), Pango (https://cov-lineages.org/), Nextstrain (https://nextstrain.org/), and the Global Initiative on Sharing Avian Influenza Data (GISAID) (https://gisaid.org/) established nomenclature systems for naming and tracking SARS-CoV-2 genetic lineages [3,4]. Variants and subvariants of this virus, which resulted in pandemic waves and millions of deaths around the world, included Alpha (B.1.1.7), Beta (B.1.351), Gamma (P.1), Delta (B.1.617.2), and Omicron (B.1.1.529, BA.1, BA.2, BA.4, and BA.5 lineages) [5]. The naming and tracking of SARS-CoV-2 genetics have been assisted by deposits of SARS-CoV-2 genomic sequences in public genome sharing databases, such as GISAID and the GeneBank of the National Center for Biotechnology Information. As of November 2022, over 13 million complete and high-coverage genomes of SARS-CoV-2 have been deposited in GISAID and are globally accessible; among them, >1500 sequences are from Saudi Arabia.

SARS-CoV-2 exhibits genetic similarities to the Chinese-originated SARS-CoV-1. It is an enveloped virus and comprises a linear, non-segmented, positive-sense single-stranded RNA genome (+ssRNA) of 8.4–12 KDa in size [1,6]. The SARS-CoV-2 genome encodes 14 open reading frames, which are responsible for the expression of approximately 29 structural and nonstructural proteins [7]. The 5’ terminal makes up about two-thirds of the viral genome and is filled with open reading frames, which encode proteins necessary for virus replication. In contrast, the 3’ terminal makes up about one-third of the genome and contains five structural proteins, including the spike protein (S), membrane protein (M), nucleocapsid protein (N), envelope protein (E), and hemagglutinin-esterase protein (HE) [1]. The spike protein is distributed on the surface of the virus and is responsible for attaching and binding to the human receptor for angiotensin-converting enzyme 2 [8]. Furthermore, the spike protein has been the target of developed vaccines and therapeutics. In addition, many studies have focused on tracking emerging mutations in the spike protein and their impacts on virus transmissibility, disease pathogenesis, and immunogenicity. For example, the P681R mutation was shown to increase virus transmissibility, and the E484K mutation was shown to affect antibody neutralization [9,10]. However, in addition to these studies on the spike protein, more research should be done to find and evaluate mutations in other virus proteins, like the N protein. The N protein plays numerous essential roles in the infection cycle of the virus. Specifically, the N protein contributes to viral RNA assembly and packaging into the riboneucleocapsid protein complex. The N protein also facilitates viral RNA replication and translation [11]. These important functions involve two conserved structural regions: (1) the N-terminal domain, representing the RNA binding domain (residues 44–174), and (2) the C-terminal domain, representing the dimerization domain (residues 255–364). These two domains are flanked by three intrinsically disordered regions: (1) the N-arm (residues 1–43), (2) a central linker region (residues 175–254), and (3) the C-tail (residues 365–419). The central linker region contains an SR-rich motif, enriched with serine and arginine residues. It connects the N-terminal domain and the C-terminal domain, with the N-arm and C-tail existing at the sides of the N-terminal and C-terminal domains [12,13,14]. Owing to the important roles of these regions in the assembly and synthesis of viral RNA, surveillance of SARS-CoV-2 should include tracking the evolution of mutations in these regions and the influences of these mutations on the characteristics of the virus. Here, we report amino acid changes in the N protein and the association of these mutations with the demographic and clinical characteristics of SARS-CoV-2-positive patients visiting the King Faisal Specialist Hospital and Research Centre (KFSHRC), a tertiary referral hospital located in Saudi Arabia which provides specialized care for, among other specialties, cancer, transplantation, and individuals who are immunocompromised.

## 2. Methods

### 2.1. Collection of Patient Samples and Demographic and Clinical Data

Nasopharyngeal swab samples that were polymerase chain reaction (PCR)-confirmed positive for SARS-CoV-2 were deidentified, coded, and obtained in viral transport media from the Microbiology Section of the Pathology and Laboratory Medicine Department at KFSHRC in Riyadh, Madinah, and Jeddah. Samples were collected from 695 patients during the Delta and Omicron variant waves from 1 April 2021 to 30 April 2022. The related clinical and demographic electronic health records were obtained from the Infection Control and Hospital Epidemiology Department at KFSHRC.

### 2.2. Whole Genome Sequencing

Unless otherwise specified, all equipment and kits for whole genome sequencing were purchased from Thermo Fisher Scientific, Waltham, MA, USA. Total viral RNA was extracted from 200 μL of viral transport media using the MagMAX™ Viral/Pathogen II Nucleic Acid Isolation Kit. Detection of viral RNA and estimation of viral load were performed using a TaqPath™ COVID-19 CE-IVD RT-PCR Kit that targets the N, spike, and open reading frame 1ab genes in SARS-CoV-2. Extracts that were identified as positive via real-time PCR were converted to cDNA using an Invitrogen SuperScript™ IV VILO™ Master Mix kit. The assays were conducted according to the manufacturer’s instructions. The cDNA was used to prepare libraries with the Ion AmpliSeq™ SARS-CoV-2 Research Panel. The preparation of the libraries included (1) amplification of the targets, (2) partial digestion of amplicons, and (3) ligation of adapters to the amplicons. For each sample, two separate amplification reactions were conducted, with primer pool 1 used in one reaction and primer pool 2 used in the other. The amplification reactions were then combined. The amplified cDNA target was then partially digested. The products were ligated with unique barcode adapters using Ion Xpress™ Barcode Adapters 1-96 Kits. Each library was purified using 45 µL (i.e., 1.5× sample volume) of the Agencourt™ AMPure™ XP Reagent (Beckman Coulter, Brea, CA, USA). Library preparation was performed according to the Ion AmpliSeq™ Library Kit Plus User Guide (MAN0006735) and following the Ion AmpliSeq™ RNA Libraries protocol. All reactions were performed using a VeritiTM 96-well Thermal Cycler. All barcoded libraries were quantified using an Ion Library TaqMan Quantitation Kit, normalized from the original libraries using nuclease-free water to 28–33 pM for the Ion 520–530 chip or to 50 pM for the Ion 540 chip, and then pooled in equal volumes based on selected Ion chip capacity prior to undergoing automated template preparation. Template preparation included emulsion PCR and then immobilization of each DNA fragment on Ion Sphere™ Particles. These cloned DNA fragments were loaded into wells of an electronic semiconductor chip (Ion 520™ Chip, Ion 530™ Chip or Ion 540™ Chip). Automatic template preparation was performed using the Ion Chef™ Instrument with the Ion 510™ & Ion 520™ & Ion 530™ Kit or with the Ion 540™ Kit. Whole genome sequencing was performed with the Ion GeneStudio™ S5 System using Ion S5 sequencing solutions from the Ion 510™ & Ion 520™ & Ion 530™ Kit-Chef or the Ion 540™ Kit-Chef.

### 2.3. Data Analysis

Torrent Suite™ Software version 5.12 (Thermo Fisher Scientific, Waltham, MA, USA) was used to analyze the sequencing data. The sequences were aligned with the Wuhan-Hu-1 reference genome (accession number MN908947.3). The de novo report of the assembled contigs into FASTA file format was performed using the AssemblerTrinity plugin (v1.2.1.0). Metrics for quality control of each sequence were analyzed with the CoverageAnalysis plugin (v5.10.0.3). Single-nucleotide variants and indel variants were called using the variantCaller plugin (v5.10.1.19). Variant call files were analyzed with the COVID19AnnotateSnpEff plugin (v1.2.1.0) to predict the effects of mutations at the nucleic acid level and at the amino acid level. FASTA files of consensus sequences were generated using the IRMAreport plugin (v1.2.1.0). Consensus sequences were analyzed with the Nextclade web tool v2.8.1 (https://nextstrain.org) to align N gene sequences from our population and compare them with the Wuhan-Hu-1 sequence.

### 2.4. Statistical Analysis

The initial number of samples sequenced was 712. Samples were checked for duplicates, and patients with identical sequencing results were removed or merged. The final number of samples included in the study was 695. Data were cleaned and analyzed using SAS, version 9.4, and Prism, version 9.0 (GraphPad). Inferential and descriptive statistics were conducted to assess clinical variables. *t* tests were used to assess continuous variables, and χ^2^ tests were used to assess categorical variables. Logistic regression was conducted to estimate the risk of intensive care unit (ICU) admission and of death. All reported *p* values were two-tailed and were considered to be statistically significant at <0.05.

### 2.5. Data Availability

The data and codes used in this study are available on request. The SARS-CoV-2 sequences were deposited on the GISAID website.

## 3. Results

### 3.1. Patient Demographic Characteristics

Samples were collected from 695 patients beginning on 1 April 2021 and ending on 30 April 2022. Most samples were collected in January 2022 (47.9%), followed by June 2021 (11.2%), and most patients visited the hospital located in Riyadh (75.3%), followed by Jeddah (13.1%), and then Madinah (11.7%). The mean (SD) age of the patients was 38.9 (18.5) years, with the youngest being 3 weeks and the eldest being 102 years. By gender, 53.5% of the patients were female and 46.5% were male. The majority of the patients were Saudi nationals (72.3%) and were non-smokers (92.6%). By the end of our study, 80.7% of patients had recovered without the need for hospitalization, 5.8% died, and the remaining patients were either discharged after recovering or still recovering in the hospital. In total, 55.7% of patients had no comorbidity, whereas 44.3% presented with comorbidities, including 22.6% who were immunocompromised, 15.5% with diabetes, 24.8% with hypertension, and 7.1% (48) with organ transplants. Most patients were symptomatic (81.6%), with only 13.7% being asymptomatic. Most patients presented with mild disease severity (83.4%), followed by Stage C (10.7%) and Stage D (5.8%) disease severity [15]. Most patients did not require hospitalization (80.0%), but some (9.5%) required a short period of hospitalization and about the same number (10.5%) required a longer period of hospitalization, although this lasted for <20 days. Of our cohort, 13.6% were admitted to the ICU. The viral load detected in most patients was moderate (cycle threshold [Ct]: 20–30) (61.8%), with 27% of patients showing high viral load (Ct < 20).

Most patients were vaccinated (58.7%), but 7.2% were unvaccinated, and the vaccination status of 34.1% was unknown. The vaccines most frequently received were from Pfizer (45.6%), followed by AstraZeneca (32.4%), with 15.0% of patients receiving vaccines from more than one company, and the rest were unknown (7.1%). Most patients received a second dose (39.5%), with 33.6% receiving only the first dose but 22.5% receiving a booster dose.

### 3.2. Variants Detected from 1 April 2021 to 30 April 2022

**Figure 1:**
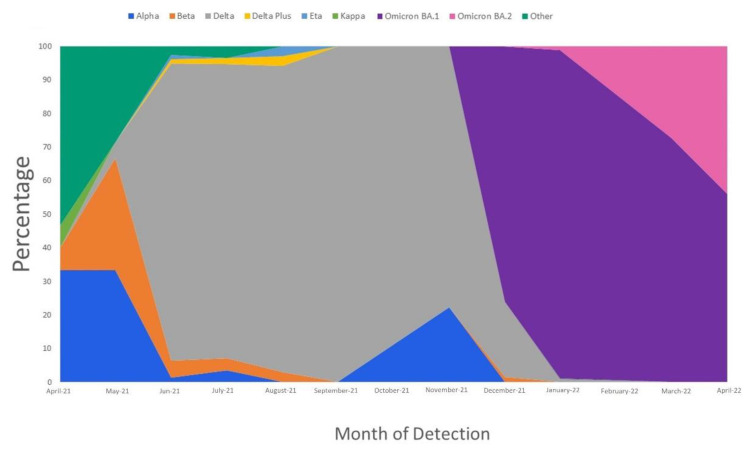
Wave distribution by month and variant detected.

The most frequently detected variants in our population were Omicron (BA.1) in 59% of the samples, followed by Delta in 26%, Beta in 3.6%, and Alpha in 3.4% (Table 1). Most cases were from samples collected during the Omicron wave (67.4%), followed by the Delta wave (32.6%) (Figure 1). The number of patients infected with Delta increased such that it was the predominant variant in June 2021 and remained in circulation in a high proportion from August to November 2021. The number of patients infected with the Delta variant then declined in December 2021 and it was replaced by infections with the Omicron BA.1 subvariant, which became the predominant variant in January 2022 and remained highly prevalent by the end of our study on 30 April 2022.

### 3.3. Amino Acid Mutations Detected in the N Protein

**Figure 2:**
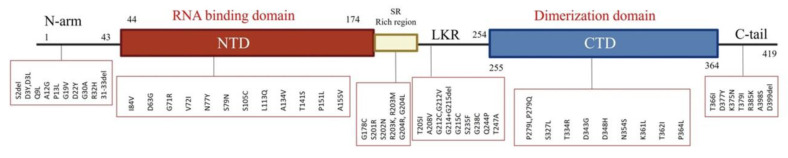
Distribution of the identified amino acid mutations across the various regions of the nucleocapsid (N) protein. NTD, N-terminal domain; LKR, linker region; and CTD, C-terminal domain.

**Figure 3:**
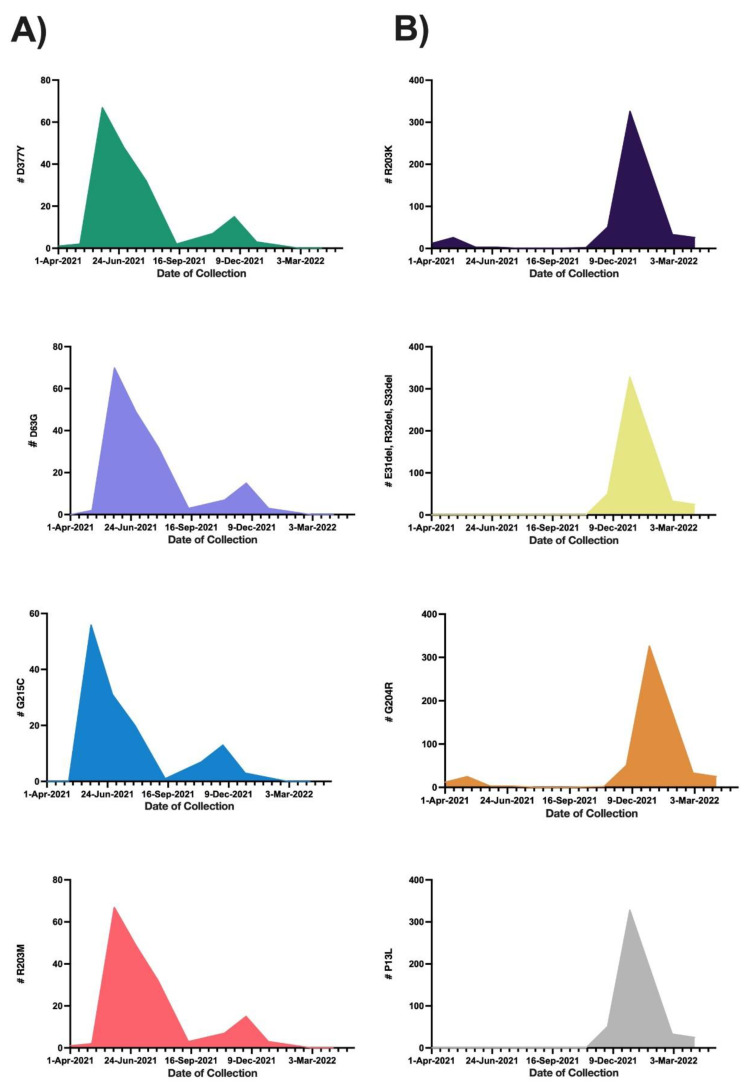
Distribution of the most frequently detected amino acid mutations in the nucleocapsid protein by date of sample collection. (**A**) shows amino acid mutations with frequencies that peaked in June 2021 and decreased in April 2022. (**B**) shows amino acid mutations with frequencies that peaked in January 2022.

Our analysis identified 60 amino acid mutations in the N protein derived from all patient samples. The most frequently mutated region within the protein was the SR-rich region, which represented 97.1% of the regions. The C-terminal domain had the fewest mutations (3.5%). A summary of the regions with amino acid mutations is given in Table 2 and shown in Figure 2. All amino acids in the N protein that showed mutations are given in Table 3 by frequency of mutation and percentage of the total sample. The most common amino acid mutations were R203K and G204R (both approximately 70%), followed by P13L, E31del, R32del, and S33del (all approximately 64%). Figure 3 shows the frequency of the most common mutations detected by sample collection date across time. Overall, these mutations showed two similar patterns: the first included D377Y, D63G, G215C, and R203M, with detection frequencies peaking in June 2021 and then decreasing in January 2022 (Figure 3A); the second pattern included R203K, E31del, R32del, S33del, G204R, and P13L, with detection frequencies peaking in January 2022 and remaining predominant at the study’s end (30 April 2022) (Figure 3B). The two consecutive mutations of R203K and G204R were observed at the beginning of the study in April 2021, but then their frequency of detection declined in late June, then re-emerged in December 2021 and peaked in January 2022.

### 3.4. Association of the Most Frequent Amino Acid Mutations in the N Protein with Patient Demographic and Clinical Characteristics

We used χ^2^ tests and *t* tests to assess the associations between the most frequent amino acid mutations and patient demographic and clinical characteristics. We selected eight of the most frequent amino acid mutations in our data, which include R203K, G204R, 31–33 del, P13L, D63G, R203M, D377Y, and G215C. In our study, patients with the most common amino acid mutation (R203K) were significantly younger than patients with the wild-type protein (*p* < 0.05), and this mutation was detected more frequently in female patients than male patients (*p* < 0.05) (Table 4). The R203K mutation showed no association with rate of hospitalization or disease severity. No significant association was detected with vaccination status (i.e., vaccinated, unvaccinated, number of doses, and booster receipt). However, for the type of vaccine, most of the patients who received the Pfizer vaccine had the R203K mutation. By contrast, most patients with the wild-type N protein and breakthrough infection received the AstraZeneca vaccine. The R203K mutation and the wild-type protein were associated with COVID-19 symptomatic patients. They were also associated with a moderate Ct value (Ct = 20–30). The mutation was detected in both the Delta and the Omicron waves. However, it was more frequent during the Omicron wave. Similar results were found for the G204R, 31–33del, and P13L mutations, except that the 31–33del and P13L mutations were only detected in the Omicron wave. Patients with the D63G mutation were significantly older in age than those with the wild-type protein (*p* < 0.05), and male patients had this mutation more frequently than female patients (*p* < 0.05). By vaccination status, no significant association was found. However, the majority of patients who received the AstraZeneca vaccination had the D63G mutation. In contrast, the Pfizer vaccination was associated with the majority of patients who had breakthrough infections with the wild-type N protein. Both the Delta and Omicron waves had the mutation, but the Delta wave had it more frequently. Results for the R203M, D377Y, and G215C mutations were similar.

The results of the analyses for the associations of the remaining most frequently detected mutations are given in Supplemental Tables S1–S7.

The eight amino acid mutations occurred at different frequencies in the different variants. R203K, G204R, 31–33 del, and P13L were detected frequently in Omicron BA.1, in approximately 59% of the variants. D63G, R203M, D377Y, and G215C were detected frequently in Delta, in approximately 18–25.6% of the variants. Since the Omicron BA.1 and Delta variants were the most common variants that harbored the selected mutations. χ^2^ tests and *t* tests were used to estimate the association of these variants with the patient’s demographic and clinical characteristics. Significant differences were detected between the two variants. Compared to the Omicron BA.1 variant, the Delta variant was linked with the oldest age, the highest number of ICU cases, the highest number of deaths, and the most severe diseases (Table 5).

### 3.5. Risk of ICU Admission or Death by Mutation

Logistic regression was used to estimate the risk of patient ICU admission or death (Table 6). For ICU admission, a lower risk was observed for females than males and for younger vs. older patients. Unvaccinated patients had a higher risk of ICU admission (odds ratio = 5.5, CI 95%: 2.7–10.8) than vaccinated patients. Patients with a high viral load (low Ct) had a higher risk of admission compared with patients who had moderate or low viral loads. Patients with comorbidities, diabetes, or who were immunocompromised all showed a significant risk of ICU admission. The results of our analyses by amino acid mutations indicated that patients with E31del, R32del, and S33del together or with R203K, G204R, and P13L were associated with reduced ICU admission risk compared with patients with the wild-type N protein. Conversely, an increased risk for ICU admission was found for patients with the amino acid mutations D63G, R203M, and D377Y. G215C showed no significant association with ICU admission and death. By N protein region, a significant risk for ICU admission was found for patients with mutations located in the central linker region and the C-tail region, whereas patients with mutations located in the N-arm showed a reduced risk.

Regarding patient death, a lower risk was observed for females vs. males and for younger vs. older patients. No statistically significant association between vaccine status and risk of death was detected. Patients with a high viral load (low Ct) showed a higher risk of death than patients with moderate or low viral loads. Patients with comorbidities or diabetes or who were immunocompromised had a significant risk of death. The results of our analyses by mutation indicated that only patients with P13L or E31del, R32del, and S33del together were associated with a reduced death risk compared with patients with the wild-type N protein. By N protein region, a significantly lower risk of death was found for patients with mutations located in the SR-rich region compared to the wild-type N protein.

## 4. Discussion

This epidemiologic surveillance study assessed the SARS-CoV-2 variants that were circulating at KFSHRC in Saudi Arabia and the evolution of N protein mutations and their association with patient characteristics and clinical data during the Delta and Omicron waves from 1 April 2021 to 30 April 2022. Our patient cohort showed an increased frequency in the detection of the Delta (B.1.617.2) and Omicron (BA.1) variants from 1 April 2021 to 31 April 2022. Delta was the predominant variant in June 2021 but was replaced by Omicron in December 2021. These findings are in agreement with reported transmission rates from other regions, such as the United States, California, Germany, and Norway, with increases in the proportion of COVID-19 cases in December 2021 and with Omicron BA.1 being the dominant circulating variant. Thus, the findings of studies in other regions and of our findings in Saudi Arabia indicate that Omicron BA.1 has higher transmissibility than Delta [16,17,18,19].

We analyzed 695 SARS-CoV-2 sequences and identified 60 mutations in the N protein of this virus. The region in the protein with the most detected mutations was the SR-rich region, and the C-terminal domain had the fewest detected mutations. The SR-rich region is an intrinsically disordered region, and most of the protein’s phosphorylation sites are located in this region. Phosphorylation is important to the function of the N protein during RNA transcription [20,21]. Thus, a mutation in this region could alter phosphorylation. Previous studies have reported numerous important phosphorylation sites in the SR-rich region, including S184, T198, S201, S202, R203, and G204 [20,22]. In the present study, we detected six mutations in four of these phosphorylation sites: S201R, S202N, R203K, R203M, G204R, and G204L. The R203K mutation was the most frequently detected, followed by the G204R mutation. Both of them were observed in the Alpha variant and re-emerged with the Omicron variant. A similar observation was reported in other countries, including the United States [23]. Following these two mutations in predominance were P13L and the E31del, R32del, and S33del mutations, which peaked in frequency in January 2022. These mutations have been reported in other geographic regions [24,25]. P13 is located in an important T-cell epitope. Hence, a mutation in this position may change the properties of the epitope and the cellular immune response against the virus [26]. The P13L mutation and the E31del, R32del, and S33del mutations are located in the N-arm region, which is reported to function in regulating RNA binding; thus, these mutations may affect the regulation of RNA binding [27]. We observed a group of mutations (D377Y, D63G, G215C, and R203M) that peaked in June 2021 and decreased in December 2021. A similar observation has been reported for the same time frame in other countries, including the United States, Iran, Indonesia, and China [28,29,30,31]. We also observed mutation sets that were associated with specific patient characteristics. For example, the R203K, G204R, 31–33 del, and P13L mutations were detected in younger and female patients. These mutations were harbored in Omicron variants that have been observed in several studies, including our current study, to be associated with females and young ages. [18,32,33].

In our cohort, patients who were unvaccinated, had a high viral load (low Ct), a comorbidity, or were immunocompromised showed a significantly higher risk of ICU admission or death. There was no significant risk of death detected according to vaccine status. A study conducted when Delta and Omicron dominated showed increased disease severity among unvaccinated individuals or persons with one or more comorbidity [34]. Other studies have found increased protection from virus infection or severe illness among individuals who received a booster and increased risk of COVID-19 disease among persons who are immunocompromised, although this risk was reduced with vaccine receipt [35,36]. The association between high viral load (low Ct) and severe COVID-19 disease remains controversial. Consistent with our results, a study conducted in Japan examining the associations between viral load, COVID-19 disease severity, and clinical observations found that viral load was significantly higher in patients with severe disease or death than in patients with mild symptoms or asymptomatic cases [37]. By contrast, a study assessing randomly selected COVID-19 cases reported no association between disease severity and viral load, although Ct values were lower for patients who were admitted without the need for supplemental oxygen than for patients who required oxygen support [38].

Our assessments of the association of detected mutations with ICU admission and death risk indicated that D63G, R203M, G215C, and D377Y mutations were observed frequently, along with the Delta variant, which is associated with the most ICU admission cases. These mutations were reported in a case of breakthrough reinfection in which the patient experienced hypoxia and hospitalization, and the mutations were significantly associated with mortality [39]. These mutations may also play a role in spreading the virus. D63G was associated with a high viral load and may enhance virus immune escape during virus replication [40]. The mutation is located in the N-terminal domain of the N protein. The N-terminal domain interacts directly with RNA to form the ribonucleocapsid protein complex. Therefore, mutations in this region may impact the life cycle of the virus [41]. R203M and D377Y may affect the binding of antibodies [42]. As mentioned, D63G, R203M, and D377Y showed a significant association with increased risk for ICU admission, but G215C showed no significant association, whereas R203K and G204R were associated with a reduced risk of ICU admission. Multiple studies found an association between these mutations and severe COVID-19 [43,44]. A discovery cohort conducted in the United States included 683 COVID-19 patients’ sequences for the estimation of three SARS-CoV-2 genes, including the N gene and its changes in correlation with hospitalization risk. The study included frequently observed mutations in outpatients versus inpatients. Four mutations were frequently observed across the patients, including three adjacent nucleotide changes (G28881, G28882, and G28883) that led to amino acid mutations (R203K and G204R) in the nucleocapsid protein. By applying a logistic regression of hospitalization status over the mutations, it was found that R203K and G204R significantly increased the risk of hospitalization. [43]. In addition, 892 SARS-CoV-2 genomes were sequenced from patients in Saudi Arabia as part of a retrospective cohort study to report SARS-CoV-2 mutations and how they might be linked to the outcomes of COVID-19 patients. R203K and G204R mutations were found in higher frequencies in Saudi Arabia. The effect of the R203K and G204R mutations on mortality, severity, and viral load was estimated using multivariable regression. The mutations were statistically significantly associated with the severity of the disease and higher viral loads among the patients [44]. R203K and G204R mutations were linked to milder disease in our study, despite the fact that we used a similar analysis to that used in these studies. This seems to be due to the different sample sizes and timing of the studies; the two aforementioned studies were carried out before the emergence of the Omicron variant. Our study also showed an association of P13L or of E31del, R32del, and S33del together with a reduced risk of ICU admission or death compared with patients who had the wild-type N protein. These mutations have been detected in Hong Kong in the Omicron variant, which is associated with mild COVID-19 disease [45]. In this study, the association of R203K, G204R, and P13L or E31del, R32del, and S33del with reduced risk of ICU admission and death agreed with the Omicron BA.1 variant that showed lower cases of ICU admission and mortality compared to Delta. P13L or E31del, R32del, and S33del together were associated with a reduced risk of death, while R203K and G204R showed no significant association with the risk of death. A German surveillance study examined the association between Omicron BA.1 and BA.2 infections and hospitalization, intensive care unit (ICU) admission, or mortality, as compared to the Delta variant. Using a multivariate logistic regression model, Omicron BA.1 and BA.2 were associated with a reduced risk of hospitalization, ICU admission, and mortality compared to Delta [18]. Consequently, both our observations and the results of the study indicate that Omicron subvariants (BA.1 and BA.2) are less likely to induce severe disease than Delta. D63G, R203M, and D377Y mutations are associated with an increased risk of ICU admission. Almost all patients with the Delta variant infection, which is also associated with severe cases and the majority of ICU admission cases, had these mutations. Out of 179 patients with a Delta infection, the mutations were present in 178 (99.4%), 175 (97.8%), and 173 (96.6%) of Delta-infected patients, respectively. These mutations were not discovered in any of the 414 patients with Omicron BA.1 infection. In contrast, The R203K, G204R, P13L, and 31–33del mutations were associated with reduced risk of ICU admission and were found in nearly all patients who had Omicron BA.1, which was associated with lower cases of ICU admission. Out of 414 patients who were infected with Omicron BA.1, R203K and G204R were detected in 412 (99.5%) of the samples and P13L and 31–33 del were found in 413 (99.7%) of the samples. However, these mutations were not observed in any patients who had the Delta variant (0 of 179 Delta infected patients).

The present study assessed the association of each region in the N protein showing mutations with risk of ICU admission or death. Among these regions, mutations in the C-tail and the central linker regions were significantly associated with ICU admission risk. The C-tail is located within the intrinsically disordered region of the N protein. This region is connected with the C-terminal domain and thus may impact C-terminal domain oligomerization [14]. We detected seven mutations in the C-tail region. Among them, D377Y was the most frequent and was associated with increased ICU admission risk. We detected nine mutations in the central linker region. Of them, the G215C mutation was not significantly associated with the risk of ICU admission or death, whereas mutations located in the N-arm region, including P13L and the E31del, R32del, and S33del mutations, showed a reduced risk of ICU admission. We found that the SR-rich region was significantly associated with low risk of death. Although the R203M mutation in this region was associated with an increased risk for ICU admission, two other mutations in the region, R203K and G204R, were associated with a reduced risk of ICU admission.

Limitations of the study: This study identified mutations located throughout the N protein and assessed the associations of these mutations with patient demographic and clinical characteristics. We found that mild and severe effects were associated with some studied mutations. However, the variants that frequently harbored these mutations, the Delta and Omicron variants, were linked to the risks of these mutations. Isolating the impact of these mutations is obviously difficult but necessary. In this study, some detected mutations with no substantial expansion during the surveillance period were not included in our analyses. We focused on the patients’ vaccination state before SARS-CoV-2 infection. However, the vaccine brand may not be associated with a specific mutation, which may have occurred in the variant before the vaccination. Furthermore, the immune status of vaccinated and unvaccinated patients against viral protein mutations was not investigated. Information on patient condition, disease severity, vaccination status, and comorbidity was not always complete, and thus misclassification may have occurred, which may impact the results of the associations. The roles of some N protein regions are still unknown and the impact of the amino acid changes on the SARS-CoV-2 life cycle is ambiguous. Thus, future research on the structure and biochemistry of the protein is needed.

In conclusion, we reported amino acid changes in the N protein of SARS-CoV-2 that may impact virus transmission, infectivity, and immune escape. We found significant associations between both amino acid mutations and the regions of the N protein harboring the mutations with patient demographic and clinical characteristics. Additional monitoring of the evolution of the genetic changes in this SARS-CoV-2 protein may assist with risk factor and therapeutic target identification as well as vaccine development and distribution. In this study, we reported several amino acid changes in the N gene that were linked to several clinical outcomes. To validate the likelihood of the association between the mutations and clinical outcomes, this study warrants additional research, such as those involving recombinant variants with particular or several amino acid changes in the N protein.

## Figures and Tables

**Table 1:** SAR-CoV-2 variants detected in patient samples collected from 1 April 2021 to 30 April 2022.

Variant	Frequency	Percentage
Omicron BA.1	414	59.6%
Delta	179	25.8%
Alpha	24	3.46%
Omicron BA.2	24	3.46%
Other	24	3.46%
Beta	23	3.30%
Delta Plus	3	0.43%
Eta	2	0.30%
Kappa	1	0.30%
Omicron	1	0.30%

**Table 2:** Regions of the nucleocapsid (N) protein with amino acid mutations in this cohort.

N Protein Region	Frequency	Percentage
N-arm (residues 1–43)	471	67.8%
NTD (RNA binding domain) (residues 44–174)	216	31.1%
SR-rich region (residues 175–204)	675	97.1%
LKR (residues 205–254)	184	26.5%
CTD (dimerization domain) (residues 255–364)	24	3.5%
C-tail (residues 365–419)	189	27.2%

Abbreviations: NTD, N-terminal domain; LKR, linker region; CTD, C-terminal domain.

**Table 3:** All detected amino acid mutations in the nucleocapsid protein by frequency and percentage of the total sample.

Amino Acid	Frequency	Percentage
R203K	489	70.36%
G204R	487	70.07%
P13L	445	64.03%
E31del	442	63.60%
R32del	442	63.60%
S33del	442	63.60%
D63G	183	26.33%
R203M	181	26.04%
D377Y	179	25.76%
G215C	132	18.99%
S235F	24	3.45%
T205I	24	3.45%
D3L	23	3.31%
I84V	22	3.17%
T362I	12	1.73%
A155V	4	0.58%
K375N	4	0.58%
A208V	3	0.43%
D343G	3	0.43%
P151L	3	0.43%
Q9L	3	0.43%
A12G	2	0.29%
A134V	2	0.29%
N354S	2	0.29%
R385K	2	0.29%
S105C	2	0.29%
S202N	2	0.29%
S2del + D3Y	2	0.29%
T334R	2	0.29%
A398S	1	0.14%
D22Y	1	0.14%
D348H	1	0.14%
D399del	1	0.14%
D3Y	1	0.14%
G178C	1	0.14%
G19V	1	0.14%
G204L	1	0.14%
G212C	1	0.14%
G212V	1	0.14%
G214 + G215del	1	0.14%
G238C	1	0.14%
G30A	1	0.14%
G71R	1	0.14%
K361L	1	0.14%
L113Q	1	0.14%
N77Y	1	0.14%
P279L	1	0.14%
P279Q	1	0.14%
P364L	1	0.14%
Q244P	1	0.14%
R32H	1	0.14%
R40C	1	0.14%
S201R	1	0.14%
S327L	1	0.14%
S79N	1	0.14%
T141S	1	0.14%
T247A	1	0.14%
T366I	1	0.14%
T379I	1	0.14%
V72I	1	0.14%

**Table 4:** Association of the nucleocapsid protein amino acid mutation R203K with patient demographic and clinical characteristics.

Characteristic	No. (%)		χ^2^ or T (*p*-Value)
	R203K Mutation	Wild Type	
Age (mean, SD), years	36.6 (18.7)	44.3 (16.8)	5.3 (<0.0001) *
Variant			
Alpha	24 (3.5)	0 (0)	672 (<0.0001) *
Beta	1 (0.1)	22 (3.2)	
Delta	0 (0)	179 (25.8)	
Delta Plus	0 (0)	3 (0.4)	
Eta	0 (0)	2 (0.3)	
Kappa	0 (0)	1 (0.1)	
Omicron	0 (0)	1 (0.1)	
Omicron BA.1	412 (59.3)	2 (0.3)	
Omicron BA.2	24 (3.5)	0 (0)	
Other	22 (3.2)	2 (0.3)	
Wave			
Delta	45 (6.5)	179 (25.8)	380 (<0.001) *
Omicron	438 (63)	33 (4.5)	
Sex			
Male	212 (30.5)	111 (16.0)	4.2 (0.04) *
Female	271 (39.0)	101 (14.5)	
Nationality			
Saudi	340 (53.5)	119 (18.7)	26.4 (<0.001) *
Non-Saudi	93 (14.7)	83 (13.1)	
Unknown = 60			
Smoking status			
Yes	36 (5.6)	12 (1.9)	0.9 (0.35)
No	408 (63.4)	188 (29.2)	
Unknown = 51			
Patient Status			
Deceased	24 (3.5)	16 (2.3)	9.3 (0.026) *
Recovered	398 (57.8)	158 (22.9)	
Hospitalized	5 (0.7)	1 (0.2)	
Released	50 (7.3)	37 (5.4)	
Unknown = 6			
Immunocompromised			
Yes	358 (53.5)	45 (6.7)	0.065 (0.80)
No	106 (23.9)	160 (23.9)	
Unknown = 26			
ICU Admission			
Yes	50 (7.4)	42 (6.2)	11.7 (0.0006) *
No	421 (62.2)	164 (24.2)	
Unknown = 18			
Comorbidity			
Yes	213 (31.8)	84 (12.5)	1.3 (0.25)
No	252 (37.6)	121 (18.1)	
Unknown = 25			
Diabetes mellitus			
Yes	59 (8.8)	45 (6.7)	9.5 (0.002) *
No	409 (60.8)	160 (23.8)	
Unknown = 22			
Hypertension			
Yes	103 (15.3)	64 (9.5)	6.5 (0.011) *
No	365 (54.2)	141 (21.0)	
Unknown = 22			
Symptoms			
Asymptomatic	30 (5.0)	2 (0.3)	10.8 (0.001) *
Symptomatic	374 (62.2)	195 (32.5)	
Unknown = 94			
Disease Severity			
Mild	374 (59.0)	155 (24.5)	16.4 (0.0003) *
Stage C	41 (6.5)	27 (4.3)	
Stage D	15 (2.4)	22 (3.5)	
Unknown = 61			
Vaccination Status			
Vaccinated	270 (59.0)	138 (30.1)	1.2 (0.27)
Unvaccinated	37 (8.1)	13 (2.8)	
Unknown = 237			
Type of vaccine			
Pfizer	141 (37.2)	45 (11.9)	81.6 (< 0.0001) *
AstraZeneca	48 (12.7)	84 (22.2)	
Mixture	58 (15.3)	3 (0.8)	
Unknown = 316			
Vaccine Dose			
Post-first	43 (11.0)	94 (24.1)	121.4 (< 0.0001) *
Post-second	125 (32.1)	36 (9.2)	
Post-booster	89 (22.8)	3 (0.8)	
Unknown = 305			
Hospitalization Duration			
None	396 (57.9)	151 (22.1)	10.1 (0.0063) *
Short (≤20 days)	38 (5.6)	27 (4.0)	
Long (>20 days)	42 (6.1)	30 (4.4)	
Unknown = 11			
Organ Transplant patient			
Yes	188 (27.7)	17 (2.5)	0.65 (0.42)
No	442 (65.2)	188 (27.7)	
Unknown = 17			
Ct Range			23.7 (<0.0001) *
High Ct > 30	53 (8.13)	20 (3.07)	
Low Ct < 20	96 (14.72)	80 (12.27)	
Moderate Ct 20–30	301 (46.17)	102 (15.64)	
Unknown = 43			

Abbreviations: ICU, intensive care unit; Ct, cycle threshold. * Significant *p* value, *p* < 0.05.

**Table 5:** Association of variants (Delta and Omicron B.A.1) with the patients’ demographic and clinical characteristics.

Characteristic	No. (%)		χ^2^ or T (*p*-Value)
	Delta N = 179	Omicron BA.1 N = 414	
Age (mean, SD), years	44.2 (16.0)	35.8 (17.8)	<0.0001 *
Sex			
Male	97 (16.3)	176 (29.7)	0.009 *
Female	82 (13.8)	238 (40.1)	
Nationality			
Saudi	96 (17.9)	292 (54.4)	<0.0001 *
Non-Saudi	74 (13.8)	75 (14.0)	
Unknown = 56			
Smoking status			
Yes	9 (1.7)	29 (5.3)	0.33
No	159 (29.1)	350 (64.0)	
Unknown = 46			
Patient Status			
Deceased	12 (2.0)	16 (2.7)	0.0009 *
Recovered	133 (22.7)	355 (60.5)	
Hospitalized	1 (0.2)	4 (0.70)	
Released	33 (5.6)	33 (5.6)	
Unknown = 6			
Immunocompromised			
Yes	37 (6.5)	77 (13.5)	0.57
No	136 (23.8)	321 (80.0)	
Unknown = 22			
ICU Admission			
Yes	34 (5.9)	32 (5.5)	<0.0001 *
No	140 (24.2)	372 (64.4)	
Unknown = 15			
Comorbidity			
Yes	70 (12.3)	171 (30.0)	0.58
No	103 (18.0)	227 (39.8)	
Unknown = 22			
Diabetes mellitus			
Yes	36 (6.8)	43 (7.5)	0.0013 *
No	137 (28.9)	358 (62.4)	
Unknown = 19			
Hypertension			
Yes	52 (9.1)	83 (14.5)	0.0153 *
No	121 (21.1)	318 (55.4)	
Unknown = 19			
Symptoms			
Asymptomatic	2 (0.40)	23 (5.6)	0.007 *
Symptomatic	163 (32.4)	315 (62.6)	
Unknown = 90			
Disease Severity			
Mild	132 (24.5)	327 (60.8)	<0.0001 *
Stage C	22 (4.1)	27 (5.0)	
Stage D	19 (3.5)	11 (2.0)	
Unknown = 55			
Vaccination Status			
Vaccinated	123 (31.1)	243 (61.4)	0.69
Unvaccinated	9 (2.3)	21 (5.3)	
Unknown = 197			
Type of vaccine			
Pfizer	38 (11.2)	130 (38.4)	<0.0001 *
AstraZeneca	78 (23.0)	34 (10.0)	
Mixture	1 (0.30)	58 (17.1)	
Unknown = 254			
Vaccine Dose			
Post-first	86 (24.6)	23 (6.6)	<0.0001 *
Post-second	30 (8.6)	122 (34.9)	
Post-booster	2 (0.60)	87 (25.4)	
Unknown = 243			
Hospitalization Duration			
None	127 (21.8)	356 (61.1)	<0.0001 *
Short (≤20 days)	24 (4.1)	24 (4.1)	
Long (>20 days)	24 (4.1)	28 (4.8)	
Unknown = 10			
Organ Transplant patient			
Yes	15 (2.6)	21 (3.6)	0.11
No	158 (27.3)	385 (66.5)	
Unknown = 14			
Ct Range			
High Ct > 30	14 (2.5)	43 (7.7)	<0.0001 *
Low Ct < 20	67 (12.0)	82 (14.7)	
Moderate Ct 20–30	90 (16.2)	261 (46.9)	
Unknown = 36			

Abbreviations: ICU, intensive care unit; Ct, cycle threshold. * Significant *p* value, *p* < 0.05.

**Table 6:** Estimated odds of ICU admission and death for patients with COVID-19 by patient characteristic and mutation.

Characteristic	ICU Admission		Death	
	OR (95% CI)	χ^2^ (*p*-Value)	OR (95% CI)	χ^2^ (*p*-Value)
Sex				
Male	1	5.2 (0.02) *	1	7.6 (0.0058) *
Female	0.59 (0.38–0.93)		0.39 (0.20–0.78)	
Age group, years				
0–19	1	125.8 (< 0.001) *	1	94.1 (<0.001) *
20–29	0.33 (0.10–1.05)		NA	
30–39	0.23 (0.067–0.78)		0.24 (0.02–2.6)	
40–49	1.22 (0.48–3.1)		1.32 (0.24–7.4)	
50–59	1.44 (0.56–3.73)		1.9 (0.34–10.7)	
60–102	10.8 (4.624.9)		18.8 (4.3–81.8)	
Nationality				
Non-Saudi	0.024 (0.003–0.177)	52.9 (<0.0001) *	0.067 (0.009–0.49)	17.4 (<0.001) *
Saudi	1		1	
Vaccination status				
Vaccinated	1	21.1 (<0.0001) *	1	3.3 (0.068)
Unvaccinated	5.5 (2.7–10.81)		2.9 (1.0–8.4)	
Type of vaccine				
Pfizer	5.4 (1.58–18.61)	12.0 (0.0025) *	6.7 (0.83–53.2)	5.5 (0.063)
AstraZeneca	1		1	
Mixture	1.45 (0.23–8.9)		2.2 (0.13–35.5)	
Vaccine dose				
Post-first	0.74 (0.28–2.00)	1.91 (0.38)	0.25 (0.049–1.36)	3.8 (0.15)
Post-second	1.32 (0.55–3.17)		0.91 (0.28–2.9)	
Post-booster	1		1	
Ct range				
High Ct > 30	0.95 (0.41–2.25)	11.8 (0.0027) *	0.64 (0.14–2.8)	10.1 (0.0065) *
Low Ct < 20	2.36 (1.4–3.8)		2.7 (1.4–5.4)	
Moderate Ct 20–30	1		1	
Hypertension				
No	1	221.99 (<0.0001) *	1	30.9 (<0.0001) *
Yes	6.2 (3.9–9.9)		6.5 (3.3–12.7)	
Diabetes mellitus				
No	1	249.7 (<0.0001) *	1	50.0 (<0.0001) *
Yes	9.6 (5.9–15.7)		11.7 (5.9–23.1)	
Comorbidity				
No	1	146.8 (<0.0001) *	NA	NA
Yes	80.6 (19.7–330.8)		NA	
Immunocompromised				
No	1	179.9 (<0.0001) *	1	64.3 (<0.0001) *
Yes	30.95 (17.1–55.9)		17.1 (7.7–38.2)	
Organ Transplant patient				
No	1	132.9 (<0.0001) *	1	10.5 (<0.001) *
Yes	93.4 (32.1–271.9)		4.4 (1.98–10.0)	
Amino acid mutation (Ref = 0)				
R203K	0.46 (0.30–0.72)	10.9 (0.0009) *		
G204R	0.45 (0.28–0.70)	12.1 (0.005) *	0.58 (0.30–1.1)	2.6 (0.10)
P13L	0.37 (0.230.58)	19.1 (<0.0001) *	0.51 (0.27–0.96)	4.2 (0.039) *
E31del, R32del, S33del	0.37 (0.24–0.59)	18.8 (<0.0001) *	0.51 (0.27–0.97)	4.1 (0.0417) *
D63G	1.83 (1.15–2.91)	6.24 (0.0125) *	1.23 (0.61–2.5)	0.33 (0.56)
R203M	1.86 (1.2–2.96)	6.6 (0.01) *	0.64 (0.33–1.23)	1.72 (0.18)
D377Y	1.89 (1.19–3.0)	7.0 (0.0081) *	1.3 (0.63–2.6)	0.44 (0.50)
G215C	0.61 (0.37–1.0)	3.52 (0.068)	0.93 (0.42–2.1)	0.036 (0.84)
Mutation region (Ref = 0)				
N-arm	0.41 (0.26–0.65)	14.9 (0.0001) *	0.62 (0.33–1.19)	1.96 (0.16)
NTD (RNA binding domain)	2.1 (1.3–3.3)	10.1 (0.0015) *	1.7 (0.9–3.2)	2.5 (0.11)
SR-rich region	0.35 (0.13–0.04)	3.7 (0.053)	0.23 (0.072–0.71)	4.9 (0.026) *
LKR	2.2 (1.4–3.5)	10.95 (0.0009) *	1.54 (0.78–3.0)	1.5 (0.22)
CTD (dimerization domain)	1.7 (0.62–4.7)	0.98 (0.32)	1.5 (0.34–6.7)	0.27 (0.61)
C-tail	1.9 (1.2–3.0)	7.2 (0.007) *	1.2 (0.57–2.3)	0.16 (0.68)

Abbreviations: Ct, cycle threshold; CTD, C-terminal domain; ICU, intensive care unit; LKR, linker region; OR odds ratio; NTD, N-terminal domain; Ref, reference. * Significant *p* value, *p* < 0.05.

## Data Availability

The data and codes presented in this study are available on request from the corresponding author. The data are not publicly available due to privacy restrictions. The SARS-CoV-2 sequences were deposited on the GISAID website.

## Supplementary Materials

The following supporting information can be downloaded at: https://www.mdpi.com/article/10.3390/microorganisms11051288/s1.
