# Supplementary material for: Association of SARS-CoV-2 Nucleocapsid Protein Mutations with Patient Demographic and Clinical Characteristics during the Delta and Omicron Waves
Source: Microorganisms. 2023 May 15;11(5):1288. doi: 10.3390/microorganisms11051288 (PMC10224071; doi:10.3390/microorganisms11051288)
Supplement: Supplementary file 1 [file microorganisms-11-01288-s001.zip › microorganisms-2356018-supplementary.pdf]

Table S1. Association of the nucleocapsid protein amino acid mutation G204R with patient demographic and clinical characteristics

**Characteristic**	**No.(%)**		**χ^2 or T**
	**G204R Mutation**	**Wild Type**	**(P-value)**
**Age (mean, SD), years**	36.6(18.7)	44.3(16.8)	5.4(<0.0001)*
**Variant**			
Alpha	24(3.5)	0(0)	665.5(<0.0001)*
Beta	1(0.1)	22(3.2)	
Delta	0(0.0)	179(25.8)	
Delta Plus	0(0.0)	3(0.4)	
Eta	0(0.0)	2(0.3)	
Kappa	0(0.0)	1(0.1)	
Omicron	0(0.0)	1(0.1)	
Omicron BA.1	412(59.3)	2(0.3)	
Omicron BA.2	24(3.5)	0(0.0)	
Other	20(2.9)	4(0.6)	
**Wave**			
Delta	43(6.19)	181(26.04)	387(<0.0001)*
Omicron	438(63.02)	33(4.75)	
**Sex**			
Male	211(38.9)	112(14.7)	3.9(0.047)*
Female	270 (30.4)	102(14.7)	
**Nationality**			
Saudi	338(53.2)	121(19.1)	24.4 (<0.0001)*
Non-Saudi	93(14.7)	83(13.1)	
Unknown=60			
**Smoking status**			
Yes	36(5.6)	12(1.9)	0.97(0.32)
No	406(63.0)	190(9.5)	
Unknown=51			
**Patient Status**			
Deceased	17(2.47)	23(3.34)	9.9(0.02)*
Recovered	159(23.08)	397(57.62)	
Hospitalized	1(0.15)	5(0.73)	
Released	37(5.37)	50(7.26)	
Unknown=6			
**Immunocompromised**			
Yes	105(15.7)	46(6.9)	0.02(0.90)
No	357(53.4)	161(24.1)	
Unknown=26			
**ICU Admission**			
Yes	49(7.2)	43(6.4)	12.8(0.0003)*
No	420(62.0)	165(24.4)	
Unknown=18			
**Comorbidity**			
Yes	122(31.5)	85(12.7)	1.3(0.26)
No	251(37.5)	122(18.2)	

Unknown=25			
**Diabetes mellitus**			
Yes	58(8.6)	46(6.8)	10.5(0.0012)*
No	408(60.6)	161(23.9)	
Unknown=22			
**Hypertension**			
Yes	102(15.2)	46(9.7)	6.9(0.0084)*
No	364(54.1)	161(21.1)	
Unknown=22			
**Symptoms**			
Asymptomatic	30(5.0)	2(0.3)	11.0(0.0009)*
Symptomatic	372(61.9)	197(32.8)	
Unknown=94			
**Disease Severity**			
Mild	373(58.8)	156(24.6)	18.6(<0.0001)*
Stage C	41(6.5)	27(4.3)	
Stage D	14(2.2)	23(3.6)	
Unknown=61			
**Vaccination Status**			
Vaccinated	270(59.0)	138(30.1)	0.29(0.6)
Unvaccinated	138(7.6)	15(3.3)	
Unknown=237			
**Type of vaccine**			
Pfizer	141(37.2)	45(11.9)	81.6(<0.0001) *
AstraZeneca	48(22.2)	84(12.7)	
Mixture	58(15.3)	3(0.80	
Unknown=316			
**Vaccine Dose**			
Post first	43(11.03)	94(24.1)	121.4(<0.0001)*
Post second	125(32.05)	36(9.23)	
Post booster	89(22.82)	3(0.77)	
Unknown=305			
**Hospitalization**			
None	151(22.1)	396(57.9)	12.4(0.002)*
Short (≤20 days)	38(5.9)	27(4.7)	
Long (>20 days)	40(5.6)	32(4.0)	
Unknown=11			
**Organ Transplant**			
Yes	30(4.4)	18(2.7)	1.2(0.28)
No	441(65.0)	189(27.9)	
Unknown=17			
**Ct Range**			
high Ct >30	53(11.2)	20(8.13)	24.4(<0.0001)*
Low Ct <20	95(14.57)	81(12.42)	
Moderate Ct 20-30	300(46.01)	103(15.8)	
Unknown=43			

Abbreviations: ICU, intensive care unit, Ct, cycle threshold. *Significant P value, P<0.05.

Table S2. Association of the nucleocapsid protein amino acid mutations E31del, R32del, and S33del with patient demographic and clinical characteristics

**Characteristic**	**No.(%)**		**χ^2 or T**
	**Del Mutation**	**Wild Type**	**(P-value)**
**Age (mean, SD), years**	36.2(18.7)	43.7(17.1)	5.4(<0.0001)*
**Variant**			
Alpha	0(0)	24(3.45)	690.7(<0.0001)*
Beta	0(0)	23(3.31)	
Delta	0(0)	179(25.76)	
Delta Plus	0(0)	3(0.43)	
Eta	0(0)	2(0.29)	
Kappa	0(0)	1(0.14)	
Omicron	0(0)	1(0.14)	
Omicron BA.1	413(59.42)	1(0.14)	
Omicron BA.2	24(3.45)	0(0)	
Other	0(0)	24(3.45)	
**Wave**			
Delta	0	224(32.2)	559.9(<0.0001)*
Omicron	437(62.9)	34(4.9)	
**Sex**			
Male	190(27.3)	133(19.1)	4.2(0.04)*
Female	247(35.5)	125(18.0)	
**Nationality**			
Saudi	310(48.8)	149(23.5)	28.9 (<0.0001)*
Non-Saudi	78(12.3)	98(15.4)	
Unknown=60			
**Smoking status**			
Yes	32(5.0)	16(2.5)	0.42(0.51)
No	369(57.3)	227(35.3)	
Unknown=51			
**Patient Status**			
Deceased	19(2.76)	21(3.05)	8.9(0.03)*
Recovered	362(52.54)	194(28.16)	
Hospitalized	4(0.58)	2(0.29)	
Released	46(6.68)	41(5.95)	
Unknown=6			
**Immunocompromised**			
Yes	93(13.9)	58(8.7)	0.11(0.73)
No	327(48.9)	191(28.6)	
Unknown=26			
**ICU Admission**			
Yes	39(5.8)	53(7.8)	19.5(<0.0001)*
No	388(57.3)	197(29.1)	
Unknown=18			
**Comorbidity**			
Yes	188(28.1)	109(16.3)	0.05(0.82)
No	233(34.8)	140(20.9)	

Unknown=25			
**Diabetes mellitus**			
Yes	51(7.6)	53(7.9)	10.3(0.0013)*
No	373(55.2)	196(29.1)	
Unknown=22			
**Hypertension**			
Yes	92(13.7)	75(11.1)	5.9(0.015)*
No	332(49.3)	174(25.9)	
Unknown=22			
**Symptoms**			
Asymptomatic	25(4.2)	7(1.2)	4.5(0.034)*
Symptomatic	337(56.1)	232(38.6)	
Unknown= 94			
**Disease Severity**			
Mild	342(53.9)	187(29.5)	21.6(<0.0001)*
Stage C	34(5.4)	34(5.4)	
Stage D	11(1.7)	26(4.1)	
Unknown=61			
**Vaccination Status**			
Vaccinated	250(54.6)	158(34.5)	1.6(0.21)
Unvaccinated	26(5.7)	24(5.2)	
Unknown= 237			
**Type of vaccine**			
Pfizer	135(35.6)	51(13.5)	111.2(<0.0001) *
AstraZeneca	34(9.0)	98(25.9)	
Mixture	59(15.6)	2(0.5)	
Unknown=316			
**Vaccine Dose**			
Post first	23(5.9)	114(29.23)	182.2(<0.0001)*
Post second	126(32.31)	35(8.97)	
Post booster	89(22.82)	3(0.77)	
Unknown=305			
**Hospitalization**			
None	364(53.2)	183(26.8)	15.9(0.0003)*
Short (≤20 days)	35(5.1)	30(4.4)	
Long (>20 days)	32(4.7)	40(5.9)	
Unknown=11			
**Organ Transplant**			
Yes	26(3.8)	22(3.2)	1.8(0.17)
No	403(59.4)	227(33.5)	
Unknown=17			
**Ct Range**			
high Ct >30	45(6.9)	28(4.3)	18.1(0.0001)*
Low Ct <20	86(13.2)	90(13.8)	
Moderate Ct 20-30	272(41.7)	131(20.1)	
Unknown=43			

Abbreviations: ICU, intensive care unit, Ct, cycle threshold. *Significant P value, P<0.05.

Table S3. Association of the nucleocapsid protein amino acid mutation P13L with patient demographic and clinical characteristics

**Characteristic**	**No.(%)**		**χ^2 or T**
	**P13L Mutation**	**Wild Type**	**(P-value)**
**Age (mean, SD), years**	36.1(18.6)	43.7(17.1)	5.5(<0.0001)*
**Variant**			
Alpha	0(0)	24(3.45)	690.7(<0.0001)*
Beta	0(0)	23(3.31)	
Delta	0(0)	179(25.76)	
Delta Plus	0(0)	3(0.43)	
Eta	0(0)	2(0.29)	
Kappa	0(0)	1(0.14)	
Omicron	1(0.14)	0(0)	
Omicron BA.1	413(59.42)	1(0.14)	
Omicron BA.2	24(3.45)	0(0)	
Other	0(0)	24(3.45)	
**Wave**			
Delta	0	224(32.2)	563.3(<0.0001)*
Omicron	438(63.0)	33(4.5)	
**Sex**			
Male	190(27.3)	133(19.1)	4.6(0.032)*
Female	248(35.7)	124(17.8)	
**Nationality**			
Saudi	311(49.0)	148(23.3)	29.4 (<0.0001)*
Non-Saudi	78(12.3)	98(15.4)	
Unknown=60			
**Smoking status**			
Yes	32(5.0)	16(2.5)	0.39(0.52)
No	370(57.5)	226(35.1)	
Unknown=51			
**Patient Status**			
Deceased	19(2.76)	21(3.05)	9.2(0.02)*
Recovered	363(52.7)	193(28.0)	
Hospitalized	4(0.58)	2(0.29)	
Released	46(6.68)	41(5.95)	
Unknown=6			
**Immunocompromised**			
Yes	93(13.9)	58(8.7)	0.15(0.70)
No	328(49.0)	190(28.4)	
Unknown=26			
**ICU Admission**			
Yes	39(5.8)	53(7.8)	19.8(<0.0001)*
No	389(57.5)	196(29.0)	
Unknown=18			
**Comorbidity**			
Yes	189(28.2)	108(16.1)	0.09(0.75)
No	233(34.8)	140(20.9)	

Unknown=25			
**Diabetes mellitus**			
Yes	51(7.6)	53(7.9)	10.5(0.0012)*
No	374(55.6)	195(29.0)	
Unknown=22			
**Hypertension**			
Yes	92(13.7)	75(11.1)	6.2(0.012)*
No	333(49.5)	173(25.7)	
Unknown=22			
**Symptoms**			
Asymptomatic	25(4.2)	7(1.2)	4.5(0.034)*
Symptomatic	337(56.1)	232(38.6)	
Unknown= 94			
**Disease Severity**			
Mild	343(54.1)	186(29.3)	21.9(<0.0001)*
Stage C	34(5.4)	34(5.4)	
Stage D	11(1.7)	26(4.1)	
Unknown=61			
**Vaccination Status**			
Vaccinated	252(55.0)	156(34.0)	1.8(0.18)
Unvaccinated	26(5.7)	24(5.2)	
Unknown= 237			
**Type of vaccine**			
Pfizer	136(35.9)	50(13.2)	112.5(<0.0001) *
AstraZeneca	34(9.0)	98(25.9)	
Mixture	59(15.6)	2(0.5)	
Unknown=316			
**Vaccine Dose**			
Post first	23(5.9)	114(29.2)	185.6(<0.0001)*
Post second	126(32.3)	35(9.0)	
Post booster	90(23.1)	2(0.5)	
Unknown=305			
**Hospitalization**			
None	365(53.4)	182(26.6)	16.2(0.0003)*
Short (≤20 days)	35(5.1)	30(4.4)	
Long (>20 days)	32(4.7)	40(5.9)	
Unknown=11			
**Organ Transplant**			
Yes	26(3.8)	22(3.2)	1.9(0.17)
No	404(59.6)	226(33.3)	
Unknown=17			
**Ct Range**			
high Ct >30	46(7.1)	27(4.1)	19.2(0.0001)*
Low Ct <20	85(13.0)	91(14.0)	
Moderate Ct 20-30	272(41.7)	131(20.1)	
Unknown=43			

Abbreviations: ICU, intensive care unit, Ct, cycle threshold. *Significant P value, P<0.05.

Table S4. Association of the nucleocapsid protein amino acid mutation D63G with patient demographic and clinical characteristics

Characteristic	No.(%)		χ^2 or T
	**D63G Mutation**	**Wild Type**	**(P-value)**
**Age (mean, SD), years**	43.9(16.1)	37.2(18.9)	4.6(<0.0001)*
**Variant**			
Alpha	0.0(0.0)	24.0(3.5)	689.8(<0.0001)*
Beta	0.0(0.0)	23.0(3.3)	
Delta	178.0(25.6)	1.0(0.1)	
Delta Plus	3.0(0.4)	0.0(0.0)	
Eta	0.0(0.0)	2.0(0.3)	
Kappa	0.0(0.0)	1.0(0.1)	
Omicron	0.0(0.0)	1.0(0.1)	
Omicron BA.1	0.0(0.0)	414.0(59.6)	
Omicron BA.2	0.0(0.0)	24.0(3.5)	
Other	0.0(0.0)	24.0(3.5)	
**Wave**			
Delta	152(21.87)	72(10.36)	300.0(<0.0001)*
Omicron	29(4.17)	442(63.6)	
**Sex**			
Male	96(13.8)	227(32.7)	4.2(0.04)*
Female	85(12.2)	287(41.3)	
**Nationality**			
Saudi	99(15.6)	360(56.7)	25.5 (<0.0001)*
Non-Saudi	73(11.5)	103(16.2)	
Unknown=60			
**Smoking status**			
Yes	8(1.2)	40(6.2)	2.5(0.11)
No	162(25.2)	434(67.4)	
Unknown=51			
**Patient Status**			
Deceased	12(1.7)	28(4.1)	9.3(0.029)*
Recovered	134(19.5)	422(61.3)	
Hospitalized	1(0.2)	5(0.7)	
Released	34(4.9)	53(7.7)	
Unknown=6			
**Immunocompromised**			
Yes	37(5.5)	114(17.0)	0.27 (0.59)
No	138(20.6)	380(56.8)	
Unknown=26			
**ICU Admission**			
Yes	34(5.0)	58(8.6)	6.6(0.01)*
No	142(21.0)	443(65.4)	
Unknown=18			
**Comorbidity**			
Yes	70(10.5)	227(33.9)	1.8(0.20)
No	105(15.7)	268(40)	

Unknown=25			
**Diabetes mellitus**			
Yes	36(5.4)	68(10.1)	4.7(0.03)*
No	139(20.6)	430(63.9)	
Unknown=22			
**Hypertension**			
Yes	51(7.6)	116(17.2)	2.4(0.12)
No	124(18.4)	382(56.8)	
Unknown=22			
**Symptoms**			
Asymptomatic	2(0.3)	30(5.0)	7.9(0.00049)*
Symptomatic	166(27.6)	403(67.1)	
Unknown=94			
**Disease Severity**			
Mild	134(21.1)	395(62.3)	12.6(0.0019)*
Stage C	22(3.5)	46(7.3)	
Stage D	19(3.0)	18(2.8)	
Unknown=61			
**Vaccination Status**			
Vaccinated	124(27.1)	284(62.0)	3.3(0.065)
Unvaccinated	9(2.0)	41(9.0)	
Unknown= 237			
**Type of vaccine**			
Pfizer	38(10.0)	148(39.1)	80.1(<0.0001)*
AstraZeneca	78(20.6)	54(14.3)	
Mixture	2(0.5)	59(15.6)	
Unknown=316			
**Vaccine Dose**			
Post first	86(22.1)	51(13.1)	111.7(<0.0001)*
Post second	31(8.0)	130(33.3)	
Post booster	2(0.5)	90(23.1)	
Unknown=305			
**Hospitalization**			
None	128(18.7)	419(61.3)	9.2(0.01)*
Short (≤20 days)	25(3.65)	40(5.85)	
Long (>20 days)	24(3.51)	48(7.02)	
Unknown=11			
**Organ Transplant**			
Yes	15(2.2)	33(4.9)	0.79(0.37)
No	160(23.6)	470(69.3)	
Unknown=17			
**Ct Range**			
high Ct >30	14(2.15)	59(9.05)	18.5(<0.0001)*
Low Ct <20	68(10.43)	108(16.56)	
Moderate Ct 20-30	91(13.96)	312(47.85)	
Unknown=43			

Abbreviations: ICU, intensive care unit, Ct, cycle threshold. *Significant P value, P<0.05.

Table S5. Association of the nucleocapsid protein amino acid mutation R203M with patient demographic and clinical characteristics

Characteristic	No.(%)		χ^2 or T
	**R203M Mutation**	**Wild Type**	**(P-value)**
**Age (mean, SD), years**	44.1(16.2)	37.2(18.9)	4.7(<0.0001)*
**Variant**			
Alpha	0(0)	24(3.45)	674.5(<0.0001)*
Beta	0(0)	23(3.31)	
Delta	175(25.18)	4(0.58)	
Delta Plus	3(0.43)	0(0)	
Eta	0(0)	2(0.29)	
Kappa	1(0.14)	0(0)	
Omicron	0(0)	1(0.14)	
Omicron BA.1	0(0)	414(59.57)	
Omicron BA.2	0(0)	24(3.45)	
Other	0(0)	24(3.45)	
**Wave**			
Delta	150(21.6)	442(63.6)	293(<0.0001)*
Omicron	29(4.2)	74(10.7)	
**Sex**			
Male	96(13.8)	227(32.7)	5.0(0.03)*
Female	83(11.9)	289(41.6)	
**Nationality**			
Saudi	97(15.3)	362(57.0)	26.9(<0.0001)*
Non-Saudi	73(11.5)	103(16.2)	
Unknown=60			
**Smoking status**			
Yes	9(1.4)	39(6.1)	1.4(0.22)
No	159(24.7)	437(67.9)	
Unknown=51			
**Patient Status**			
Deceased	12(1.74)	28(4.06)	9.8(0.02)*
Recovered	132(19.16)	424(61.54)	
Hospitalized	1(0.15)	5(0.73)	
Released	34(4.93)	53(7.69)	
Unknown=6			
**Immunocompromised**			
Yes	37(5.5)	114(17.0)	0.19 (0.67)
No	136(20.3)	382(57.1)	
Unknown=26			
**ICU Admission**			
Yes	34(8.6)	58(5.0)	7.1(0.0079)*
No	140(20.7)	445(65.7)	
Unknown=18			
**Comorbidity**			
Yes	70(10.5)	227(33.9)	1.4(0.23)

No	103(15.4)	270(40.3)	
Unknown=25			
**Diabetes mellitus**			
Yes	37(5.5)	67(10.0)	6.3(0.012)*
No	136(21.2)	433(64.3)	
Unknown=22			
**Hypertension**			
Yes	52(7.7)	115(17.1)	3.4(0.064)
No	121(18.0)	385(57.2)	
Unknown=22			
**Symptoms**			
Asymptomatic	2(0.3)	30(5.0)	7.6(0.0057)*
Symptomatic	163(27.1)	406(67.6)	
Unknown= 94			
**Disease Severity**			
Mild	132(20.82)	397(62.62)	13.1(0.0014)*
Stage C	22(3.47)	46(7.26)	
Stage D	19(3)	18(2.84)	
Unknown=61			
**Vaccination Status**			
Vaccinated	121(26.4)	287(62.7)	2.0(0.15)
Unvaccinated	10(2.2)	40(8.7)	
Unknown= 237			
**Type of vaccine**			
Pfizer	37(9.8)	149(39.3)	77.1(<0.0001)*
AstraZeneca	76(20.1)	56(14.8)	
Mixture	2(0.5)	59(15.6)	
Unknown=316			
**Vaccine Dose**			
Post first	83(21.28)	54(13.85)	104.3(<0.0001)*
Post second	31(7.95)	130(33.33)	
Post booster	2(0.51)	90(23.08)	
Unknown=305			
**Hospitalization**			
None	126(18.4)	421(61.6)	9.8(0.007)*
Short (≤20 days)	25(3.7)	40(5.9)	
Long (>20 days)	24(3.5)	48(7.0)	
Unknown=11			
**Organ Transplant**			
Yes	15(4.9)	33(2.2)	1.2(0.28)
No	158(23.3)	472(69.6)	
Unknown=17			
**Ct Range**			
high Ct >30	58(8.9)	15(2.3)	15.9(0.0003)*
Low Ct <20	110(16.9)	66(10.12)	
Moderate Ct 20-30	313(48.0)	90(13.8)	
Unknown=43			

Abbreviations: ICU, intensive care unit, Ct, cycle threshold. *Significant P value, P<0.05.

Table S6. Association of the nucleocapsid protein amino acid mutation D377Y with patient demographic and clinical characteristics

**Characteristic**	**No.(%)**		**χ^2 or T**
	**D377Y Mutation**	**Wild Type**	**(P-value)**
**Age (mean, SD), years**	44.0(16.2)	37.2(18.9)	
**Variant**			
Alpha	0(0.0)	24(3.5)	664(<0.0001)*
Beta	0(0.0)	23(3.3)	
Delta	173(24.9)	6(0.9)	
Delta Plus	3(0.4)	0(0.0)	
Eta	0(0.0)	2(0.3)	
Kappa	1(0.1)	0(0.0)	
Omicron	0(0.0)	1(0.1)	
Omicron BA.1	0(0.0)	414(59.6)	
Omicron BA.2	0(0.0)	24(3.5)	
Other	0(0.0)	24(3.5)	
**Wave**			
Delta	149(10.8)	75(10.8)	293.4(<0.001)*
Omicron	28(4.0)	443(63.7)	
**Sex**			
Male	95(13.7)	228(32.8)	4.2(0.026)*
Female	82(11.8)	290 (41.7)	
**Nationality**			
Saudi	97(15.3)	362(57.0)	24.1 (<0.0001)
Non-Saudi	71(11.2)	105(16.5)	
Unknown=60			
**Smoking status**			
Yes	8(1.2)	40(6.2)	2.2(0.133)
No	158(24.5)	438(68.0)	
Unknown=51			
**Patient Status**			
Deceased	12(1.74)	28(4.06)	10.4(0.0157)*
Recovered	130(18.87)	426(61.83)	
Hospitalized	1(0.15)	5(0.73)	
Released	34(4.93)	53(7.69)	
Unknown=6			
**Immunocompromised**			
Yes	134(20.0)	114(17.0)	0.11(0.73)
No	106(23.9)	384(57.4)	
Unknown=26			
**ICU Admission**			
Yes	34(5.0)	58(8.6)	7.5(0.006)*
No	138(20.4)	447(66.0)	
Unknown=18			
**Comorbidity**			
Yes	69(10.3)	228(34.0)	1.5(0.23)
No	102(15.2)	271(40.5)	
Unknown=25			
**Diabetes mellitus**			
Yes	36(5.4)	68(10.1)	5.5 (0.02)*
No	135(20.1)	434(64.5)	
Unknown=22			
**Hypertension**			

Yes	50(7.4)	117(17.4)	2.4(0.12)
No	121(18.0)	385(57.2)	
Unknown=22			
**Symptoms**			
Asymptomatic	2(0.3)	30(5.0)	7.5(0.0006)*
Symptomatic	162(27.0)	407(67.7)	
Unknown= 94			
**Disease Severity**			
Mild	130(20.5)	399(62.93)	13.7(0.0011)*
Stage C	22(3.47)	46(7.26)	
Stage D	19(3)	18(2.84)	
Unknown=61			
**Vaccination Status**			
Vaccinated	120(28.2)	288(62.9)	2.9(0.09)
Unvaccinated	9(2.0)	41(9.0)	
Unknown= 237			
**Type of vaccine**			
Pfizer	38(10.0)	148(39.1)	73.6(<0.0001)*
AstraZeneca	75(19.8)	57(15.0)	
Mixture	2(0.5)	59(15.6)	
Unknown=316			
**Vaccine Dose**			
Post first	84(21.54)	53(13.59)	108.3(<0.0001)*
Post second	30(7.69)	131(33.59)	
Post booster	2(0.51)	90(23.08)	
Unknown=305			
**Hospitalization**			
None	124(18.13)	423(61.84)	10.4(0.005)*
Short (≤20 days)	25(3.65)	40(5.85)	
Long (>20 days)	24(3.51)	48(7.02)	
Unknown=11			
**Organ Transplant**			
Yes	14(5.0)	34(5.0)	0.65(0.42)
No	157(23.2)	473(69.8)	
Unknown=17			
**Ct Range**			
high Ct >30	14(2.15)	59(9.05)	15.5(0.004)*
Low Ct <20	65(9.97)	111(17.02)	
Moderate Ct 20-30	90(13.8)	313(48.01)	
Unknown=43			

Abbreviations: ICU, intensive care unit, Ct, cycle threshold. *Significant P value, P<0.05.

Table S7. Association of the nucleocapsid protein amino acid mutation G215C with patient demographic and clinical characteristics

Characteristic	No.(%)		χ^2 or T
	**G215C Mutation**	**Wild Type**	**(P-value)**
**Age (mean, SD), years**	44.7(16.6)	37.6(18.6)	4.3(<0.0001)*
**Variant**			
Alpha	0(0)	24(3.5)	456.6(<0.0001)*
Beta	0(0)	23(3.3)	
Delta	128(18.42)	51(7.3)	
Delta Plus	3(0.4)	0()	
Eta	0(0)	2(0.3)	
Kappa	0(0)	1(0.1)	
Omicron	0(0)	1(0.1)	
Omicron BA.1	0(0)	414(59.5)	
Omicron BA.2	0(0)	24(3.5)	
Other	0(0)	24(3.5)	
**Wave**			
Delta	107(15.4)	117(16.8)	180.7(<0.0001)*
Omicron	24(3.4)	447(64.3)	
**Sex**			
Male	80(11.5)	243(35.0)	13.8(0.0002)*
Female	51(7.3)	321(46.2)	
**Nationality**			
Saudi	64	395(32.8)	32.9(<0.0001)*
Non-Saudi	60	116	
Unknown=60			
**Smoking status**			
Yes	9(1.4)	39(6.1)	0.004(0.94)
No	114(17.7)	482(74.8)	
Unknown=51			
**Patient Status**			
Deceased	8(8)	32(4.64)	10.7(0.0135)*
Recovered	96(96)	460(66.76)	
Hospitalized	0(0)	6(0.87)	
Released	27(27)	60(8.71)	
Unknown=6			
**Immunocompromised**			
Yes	27(4.0)	124(18.5)	0.19(0.65)
No	101(15.1)	417(62.3)	
Unknown=26			
**ICU Admission**			
Yes	24(3.6)	68(10.0)	3.6(0.058)
No	104(15.4)	481(71.1)	
Unknown=18			
**Comorbidity**			
Yes	51(7.6)	246(37.7)	1.3(0.25)

No	77(11.5)	296(44.2)	
Unknown=25			
**Diabetes mellitus**			
Yes	24(3.6)	80(11.9)	1.3(0.25)
No	104(15.5)	465(69.1)	
Unknown=22			
**Hypertension**			
Yes	37(5.5)	130(19.3)	1.4(0.23)
No	91(13.5)	415(61.7)	
Unknown=22			
**Symptoms**			
Asymptomatic	2(0.3)	30(5.0)	4.3(0.039)*
Symptomatic	122(20.3)	447(74.4)	
Unknown= 94			
**Disease Severity**			
Mild	99(15.62)	430(67.82)	7.4(0.0249)*
Stage C	18(2.84)	50(7.89)	
Stage D	13(2.05)	24(3.79)	
Unknown=61			
**Vaccination Status**			
Vaccinated	91(19.9)	317(69.2)	5.5(0.0186)*
Unvaccinated	4(0.9)	46(10.0)	
Unknown= 237			
**Type of vaccine**			
Pfizer	29(7.7)	157(41.4)	45.5(<0.0001)*
AstraZeneca	55(14.5)	77(20.3)	
Mixture	2(0.5)	59(15.6)	
Unknown=316			
**Vaccine Dose**			
Post first	68(17.44)	69(17.69)	89.3(<0.0001)*
Post second	19(4.87)	142(36.41)	
Post booster	2(0.51)	90(23.08)	
Unknown=305			
**Hospitalization**			
None	93(13.6)	454(66.4)	7.8(0.02)*
Short (≤20 days)	20(2.9)	45(6.6)	
Long (>20 days)	16(2.3)	56(8.2)	
Unknown=11			
**Organ Transplant**			
Yes	10(1.5)	38 (5.6)	0.13(0.72)
No	118(17.4)	512(75.5)	
Unknown=17			
**Ct Range**			
high Ct >30	12(1.84)	61(9.36)	14.4(0.0007)*
Low Ct <20	50(7.67)	126(19.33)	
Moderate Ct 20-30	61(9.36)	342(52.45)	
Unknown=43			

Abbreviations: ICU, intensive care unit, Ct, cycle threshold. *Significant P value, P<0.05.
